# Germline dependence in protein language model retrieval of adaptive immune receptors

**DOI:** 10.1093/bib/bbag614

**Published:** 2026-11-07

**Authors:** Suyue Wang, Ayan Sengupta, Songling Li, Daron M Standley

**Affiliations:** Laboratory of Genome Informatics, Research Institute for Microbial Diseases, The University of Osaka, 3-1 Yamadaoka, Suita 565-0871, Japan; MyImmune Corporation, 251 Little Falls Drive, Wilmington, DE 19808, United States; Laboratory of Genome Informatics, Research Institute for Microbial Diseases, The University of Osaka, 3-1 Yamadaoka, Suita 565-0871, Japan; Laboratory of Genome Informatics, Research Institute for Microbial Diseases, The University of Osaka, 3-1 Yamadaoka, Suita 565-0871, Japan; MyImmune Corporation, 251 Little Falls Drive, Wilmington, DE 19808, United States

**Keywords:** protein language models, immune receptor repertoires, antigen-specificity retrieval, sequence alignment, germline bias, benchmark data leakage

## Abstract

Protein language models (PLMs) are increasingly used to represent adaptive immune receptors, yet their advantage over classical sequence comparison remains unclear. We benchmarked general and receptor-specific PLMs against classical sequence-comparison methods for antigen-specific nearest-neighbor retrieval across four B- and T-cell receptor databases. Using CDR3 sequences alone, classical methods consistently outperformed general PLMs: BLOSUM62 and Levenshtein exceeded ESM2 variants by 7–12 percentage points in retrieval accuracy. Among receptor-specific models, only SCEPTR and, on one database, TCR2vec matched these baselines, although both additionally encoded V-gene information. AntiBERTa2 and IgBert did not close the performance gap. Using full variable-domain sequences, alignment and PLM performance converged. Germline-reversion, region-masking, and V-gene control analyses indicated that this convergence largely reflected conserved germline-derived context, particularly V-gene identity, with limited evidence for improved extraction of antigen-specific information from the CDRs. Germline dependence also extended to SCEPTR and TCR2vec. Heavily mutated HIV-1 antibodies provided a setting-specific exception to this pattern. Random train-test splits yielded retrieval accuracy 15–28 percentage points higher than clone-aware splits, consistent with optimistic performance estimates due to clonal leakage. These findings emphasize the need for strong sequence-comparison baselines and explicit germline controls when evaluating immune-receptor representations and support clone-aware splitting as a minimum benchmark standard.

## Introduction

Adaptive immunity relies on two classes of receptors, B-cell receptors (BCRs, antibodies) and T-cell receptors (TCRs), to recognize antigens. Identifying receptors that recognize the same antigen or epitope is a central challenge in computational immunology, with applications in vaccine design, therapeutic antibody discovery, immune monitoring, and repertoire-based disease diagnostics.

A fundamental task is “antigen-specific receptor retrieval”: given a query receptor sequence, identify reference receptors known to bind the same antigen. Operationally, this asks whether receptor sequence similarity recovers shared antigen specificity. This nearest-neighbor formulation has a long lineage in TCR analysis [1, 2] and has recently been used to evaluate learned receptor embeddings [3]. We evaluate retrieval using Recall@1 (R@1), the fraction of queries whose top-ranked match shares the query’s antigen or epitope label. Performance depends on both the similarity metric and sequence representation, from the third complementarity-determining region (CDR3) to the full variable domain.

Traditional methods compare receptor sequences by alignment, using BLOSUM62 [4] or edit distance. Protein language models (PLMs), by contrast, encode sequences as embeddings compared by vector similarity. Immune receptors, however, differ from typical proteins. Their highly variable CDR3 is generated by V(D)J recombination, with junctional insertions and deletions, and BCRs are further diversified by somatic hypermutation (SHM). Whether PLMs, trained largely on evolutionarily related proteins, can faithfully represent this non-evolutionary diversity remains unclear.

Recent studies have raised similar concerns. Germline bias has been reported across antibody-specific and, to a lesser extent, general PLMs [5]. Zero-shot PLM embeddings also underperform handcrafted representations or sequence alignment for TCR specificity unless retrained with TCR-specific objectives [3, 6, 7]. In parallel, data leakage has been shown to inflate performance in protein-interaction and immune-receptor benchmarks [8, 9].

We therefore ask three questions. Do frozen, zero-shot PLMs outperform sequence alignment for antigen-specific retrieval? How does this comparison depend on receptor representation? And how should train–test splitting account for receptor similarity to avoid optimistic performance estimates?

To answer these questions, we benchmark widely used PLMs, including ESM2, ESM-C, and receptor-specific models, against classical alignment across four immune-receptor databases spanning BCRs and TCRs and representations from the CDR3 to the full variable domain.

Our results reveal a consistent pattern across receptor types and databases. Alignment outperforms every general zero-shot PLM at the CDR3, whereas the two approaches converge at the full variable domain. Our analyses indicate that this convergence largely reflects dependence on germline-associated framework sequence, which can act as a benchmarking shortcut, rather than demonstrably improved representation of antigen-binding features. Zero-shot PLMs instead provide additional signal mainly for heavily hypermutated HIV-1 envelope antibodies. We further show that clone-unaware train-test splitting yields R@1 values 15–28 percentage points (pp) higher, motivating clone-aware evaluation as a minimum reporting standard.

These findings also provide a practical caution for immune-receptor benchmarking: improvement from CDR3-only to full-sequence PLM input should not automatically be interpreted as learning sophisticated antigen-specific context, because the gain can instead arise from a simple, low-dimensional germline (V-gene) signal. We therefore provide practical recommendations for benchmark design and reporting, including controls for germline-associated signals and clone-aware splitting (Supplementary Box S1).

## Materials and methods

We benchmarked sequence-alignment methods against PLM embeddings for antigen-specific receptor retrieval across four immune receptor databases and five receptor representations, using clone-aware splitting throughout.

### Benchmark overview

Evaluation followed a nearest-neighbor retrieval paradigm. For each seed (subsection Clone-aware splitting, seeds and statistical analysis), receptor sequences were split into a reference database (~80%) and a query set (~20%). Each method ranked all reference sequences for every query by similarity and was evaluated by Recall@1 (R@1).

Top-score ties, common in CDR3 retrieval because identical CDR3s yield identical alignment scores and embeddings, were resolved by expected R@1 (fractional credit equal to the proportion of tied candidates sharing the query label), making the metric deterministic. Tie statistics and sensitivity analyses are reported in the Supplementary Methods.

### Datasets and antigen labels

We used four publicly available immune receptor databases annotated with antigen or epitope labels. All datasets underwent the same pipeline: removal of short or nonstandard sequences, antigen-name standardization, retention of the most frequent labels, and clone-aware 80:20 splitting over 20 random seeds (subsection Clone-aware splitting, seeds and statistical analysis). Distinct receptors with identical CDR3s were retained, with ties handled as above. Dataset compositions are summarized in Supplementary Table S2; processing details are provided in the Supplementary Methods.

The Immune Epitope Database (IEDB) [10] contributed antibody heavy chains under the 10 most frequent protein antigens (~675 sequences/seed); sequences were renumbered with ANARCI [11] and trimmed to the variable domain. VDJdb [12] contributed TCRβ CDR3s under 10 epitope labels (~1000/seed). SAbDab [13] contributed paired antibodies under the five most frequent antigen labels (~310/seed). McPAS-TCR [14] contributed TCRs under 10 epitope labels (~1000/seed).

Full-length TCRβ sequences were reconstructed by grafting CDR3s onto IMGT germline V/J templates [15]; full-variable-domain analyses are therefore interpreted as directional. Download dates, label lists, and train/test sizes are provided in the Supplementary Methods.

To assess possible pretraining leakage, BCR CDR3s were searched against UniRef90 with BLAST. Seven sequences matched known broadly neutralizing antibodies, but leave-one-out analysis showed negligible effects on ESM2 performance (subsection A directional exception: PLMs lead on the most hypermutated antibodies, Supplementary Note S2.3).

### Receptor representations

We evaluated five receptor representations: CDR3, paratope, full variable domain, VJ-clonotype, and V-clonotype.

The first three progressively increase sequence context, from CDR3 alone (the most variable receptor region and principal determinant of antigen specificity [2, 16]) to the paratope (concatenated CDR1–3, the antigen-binding loops [17]) and full variable domain (~120 aa, including framework regions FW1–FW4). The latter two retain the CDR3 but restrict retrieval to receptors sharing the same V/J genes (VJ-clonotype) or V gene alone (V-clonotype), the standard clonotype definition [18]; queries lacking a matching reference receive an R@1 of zero.

V/J assignments were taken from database annotations (IEDB/SAbDab: ANARCI-assigned; VDJdb/McPAS-TCR: deposited annotations), and CDR3 boundaries followed IMGT numbering [11, 15]. Supplementary Table S12 summarizes the information isolated by each representation.

### Methods compared

We compared sequence-alignment methods (Levenshtein distance, Smith–Waterman alignment with BLOSUM62, and BLAST), the domain-specific metric TCRdist, and PLMs.

Alignment scores were computed with parasail [19], normalized by the longer sequence; gap parameters are given in the Supplementary Methods. BLAST [20] confirmed that the alignment advantage was not specific to BLOSUM62 (Supplementary Table S10).

TCRdist [1, 21] was computed with tcrdist3 using IMGT-defined CDR1, CDR2, CDR2.5 (the germline, V-gene-encoded loop between CDR2 and CDR3), and CDR3 distances from paired alpha–beta TCRs.

PLMs included general-purpose ESM2-150M, ESM2-650M, ESM2-3B [22], and ESM-C-300M [23]; antibody-specific AntiBERTy [24], AbLang [25], AntiBERTa2 [26], and IgBert [27]; and TCR-specific TCR-BERT [28]. AntiBERTa2 and IgBert were included to assess whether newer antibody-specific architectures reduce the CDR3 performance gap. Unless stated otherwise, embeddings were extracted from the final hidden layer without task-specific training (zero-shot), mean-pooled, and compared by cosine similarity. Classification (CLS) pooling results are reported in the Supplementary Material.

Two contrastively or similarity-pretrained receptor-specific TCR models, SCEPTR [3] and TCR2vec [29], were evaluated separately (subsection Generality and robustness). TCR2vec was tested in both released variants: the full-length V–CDR3–J model and the CDR3-only model (CDR3vec), allowing assessment of V/J context.

### Clone-aware splitting, seeds, and statistical analysis

To prevent clonal leakage, a recognized failure mode in immune-receptor benchmarking [30], receptors whose CDR3s shared ≥95% normalized Levenshtein similarity were clustered into single-linkage connected components and assigned entirely to the reference (~80%) or query (~20%) partition. Splitting was repeated over 20 random seeds, and reported R@1 values are seed means. Because the underlying sequence set remained fixed, variation across seeds reflects train–test partitioning rather than biological sampling. The 95% threshold is a practical proxy for clonal relatedness; robustness was assessed from 0.85 to 0.99 (subsection Generality and robustness).

Confidence intervals were estimated by nested bootstrap over seeds and queries (1000 replicates) [31]. Effect sizes were quantified with Hedges’ *g*, and practical equivalence for BCR full-variable-domain comparisons was tested using two one-sided tests (TOST) [32] with Δ = 0.05 R@1. Recall@5 and mean reciprocal rank (MRR) produced the same method ranking. Additional statistical details are provided in the Supplementary Methods and Supplementary Table S13.

## Results

We first show that clone-aware splitting is necessary for reliable evaluation of clone-novel retrieval (subsection Clone-aware splitting is a prerequisite), then compare alignment and PLMs across a representation ladder from CDR3 to the full variable domain. Alignment leads on CDR3 (subsection Alignment outperforms PLMs on CDR3-only retrieval), the two method families converge at full length through dependence on germline-associated framework context (subsection At full length, PLMs and alignment converge through a germline-associated signal), and PLMs lead only for receptors that diverge strongly from germline (subsection A directional exception: PLMs lead on the most hypermutated antibodies). Subsection Generality and robustness extends the analysis to TCR-specific methods, paired-chain retrieval, and robustness.

### Clone-aware splitting is a prerequisite

We first quantified the effect of conventional random train–test splitting. The quantity we wish to estimate is retrieval accuracy on query receptors that are not clonally related to any reference receptor, the situation faced when annotating genuinely novel clonotypes; we call this the clone-novel target. Random splitting estimates a different and easier quantity because the query and reference partitions can contain clonally related near-duplicates, and therefore reports optimistically high accuracy (Fig. 1). In a matched 20-seed comparison, random splitting increased ESM2-150M BCR Recall@1 from 0.353 to 0.632 at the CDR3 (+27.9 pp) and from 0.507 to 0.674 at the full variable domain (+16.6 pp). Similar increases occurred for alignment methods (BLOSUM62: +20.4 and + 18.7 pp, respectively). Across methods and representations, the gap between random-split and clone-aware accuracy ranged from ~15 to 28 pp, indicating clonal leakage rather than method-specific behavior. Because this gain accrues only to leaked query–reference clone pairs, we describe random-split accuracy as optimistic with respect to this clone-novel target rather than simply “biased” or “inflated,” and reserve “leakage” for the mechanism. We therefore use clone-aware splitting throughout and recommend it as a minimum standard for immune-receptor retrieval benchmarks.

**Figure 1:**
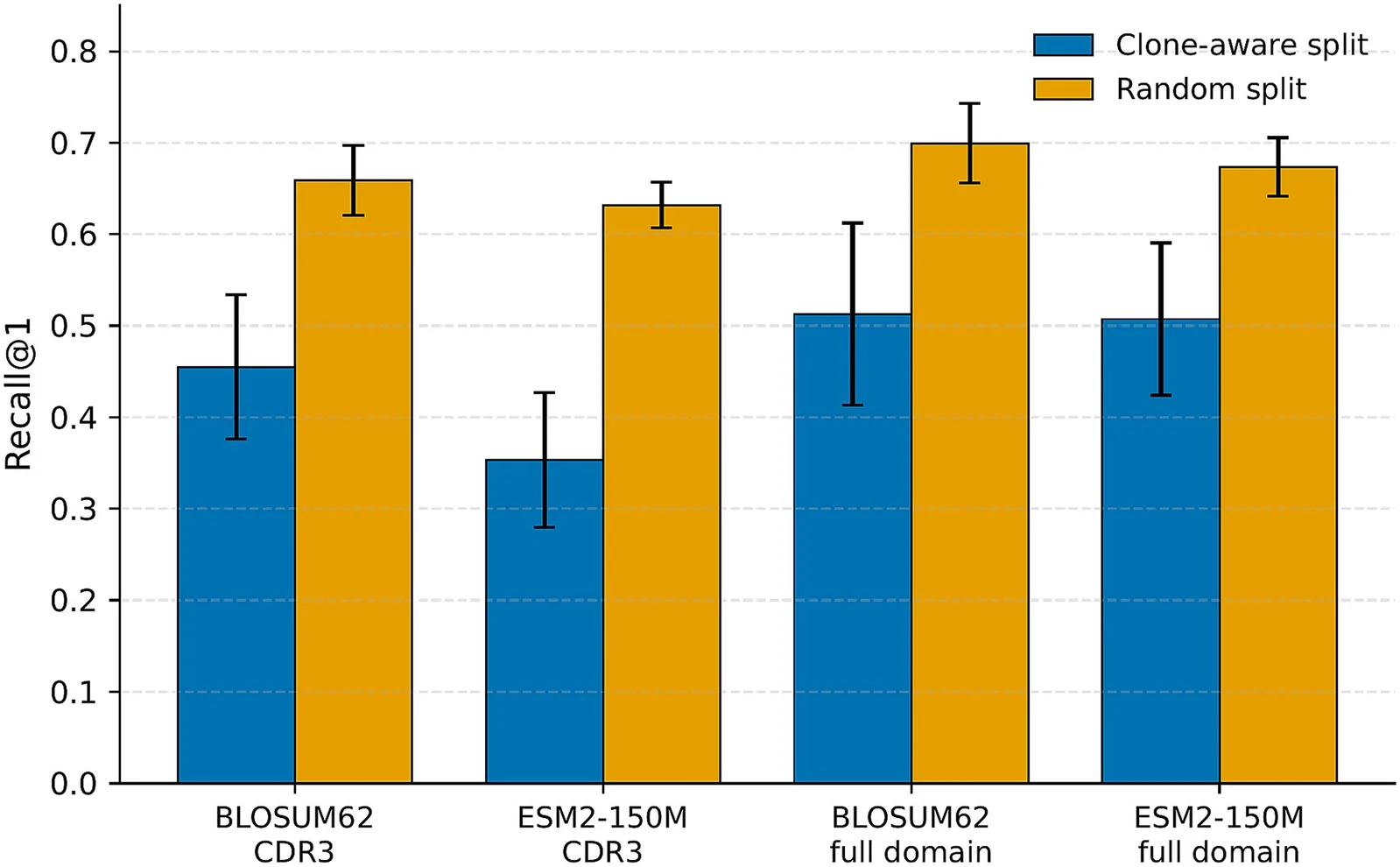
Random splits yield optimistic retrieval estimates for immune receptors. Matched 20-seed BCR comparison: random stratified splits overestimate Recall@1 relative to clone-aware splits at both CDR3-only (CDR3) and full variable domain representations, for both alignment (BLOSUM62) and the PLM (ESM2-150M), a method-general effect, not specific to PLMs (per-method gaps in subsection Clone-aware splitting is a prerequisite). Error bars: SD across 20 seeds.

### Alignment outperforms PLMs on CDR3-only retrieval

On CDR3-only inputs, classical sequence comparison achieved the highest retrieval across all four datasets (Table 1; Fig. 2). BLOSUM62 exceeded ESM2-150M (the smallest and most widely used ESM2 model, used here as the primary PLM comparator) by 10.2 pp on BCR, 9.4 pp on VDJdb TCR, 7.2 pp on SAbDab, and 11.5 pp on McPAS-TCR. Levenshtein closely tracked BLOSUM62, and no ESM2 variant (150M–3B) surpassed either alignment method; even the best ESM2 model remained 6.8–8.7 pp behind. An independent BLAST+ baseline (blastp-short) gave the same conclusion, ranking just below the best alignment method (0.6–2.9 pp) while outperforming every ESM2 variant (Supplementary Table S10). These gaps exceeded each dataset’s minimum detectable effect (~2–5.5 pp Recall@1 at 80% power), making insufficient statistical power an unlikely explanation. Effect sizes were uniformly large (paired Hedges’ *g* 1.04–3.72), and the ranking was unchanged under every tie-handling convention (Supplementary Methods). The same alignment-over-PLM ordering held under Recall@5 and MRR: BLOSUM62 led every PLM at the CDR3 on all four databases, while the methods converged into a narrow band at the full variable domain, confirming that the conclusion does not depend on Recall@1 (Supplementary Table S13).

**Table 1:** Main zero-shot retrieval Recall@1. Two alignment methods (Levenshtein, BLOSUM62) and three ESM2 variants (150M, 650M, 3B). Bold marks the highest value in each row, meaning the highest Recall@1 for each database. Values are 20-seed nested-bootstrap means of expected R@1 (order-independent tie handling; see subsection Benchmark overview). BCR is shown at the CDR3 input, the all-CDR paratope, and the full variable domain; the single highest BCR value in the table is an alignment method at the paratope (Levenshtein 0.550, the antigen-binding loops with no framework). At the BCR full variable domain, the five methods are close (R@1 range 0.508–0.541, ~3.3 pp spread), most pairs TOST-equivalent at Δ = 0.05 with the best-performing ESM2 (ESM2-650M) edging marginally above the weakest alignment method; see subsection At full length, PLMs and alignment converge through agermline-associated signal. VDJdb TCR, SAbDab, and McPAS-TCR are shown at CDR3 as cross-database CDR3-level replication; their full representation ladders (CDR3 to full variable domain) are in Supplementary Table S1, and the paired-chain TCR ladder, including the purpose-built TCRdist (R@1 = 0.515, significantly above every other method), is in Table 2 (subsection Generality and robustness). Domain-specific PLMs (AntiBERTy, AbLang, TCR-BERT) and ESM-C-300M are reported in Supplementary Table S1.

Dataset	Representation	Levenshtein	BLOSUM62	ESM2-150M	ESM2-650M	ESM2-3B
BCR (IEDB)	CDR3	0.446	**0.459**	0.357	0.372	0.350
	Paratope (all CDRs)	**0.550**	0.538	0.445	0.417	0.446
	Full variable domain	0.508	0.522	0.514	**0.541**	0.528
VDJdb TCR	CDR3	0.355	**0.375**	0.282	0.304	0.307
SAbDab	CDR3	0.351	**0.358**	0.286	0.273	0.259
McPAS-TCR	CDR3	0.360	**0.387**	0.272	0.282	0.305

**Figure 2:**
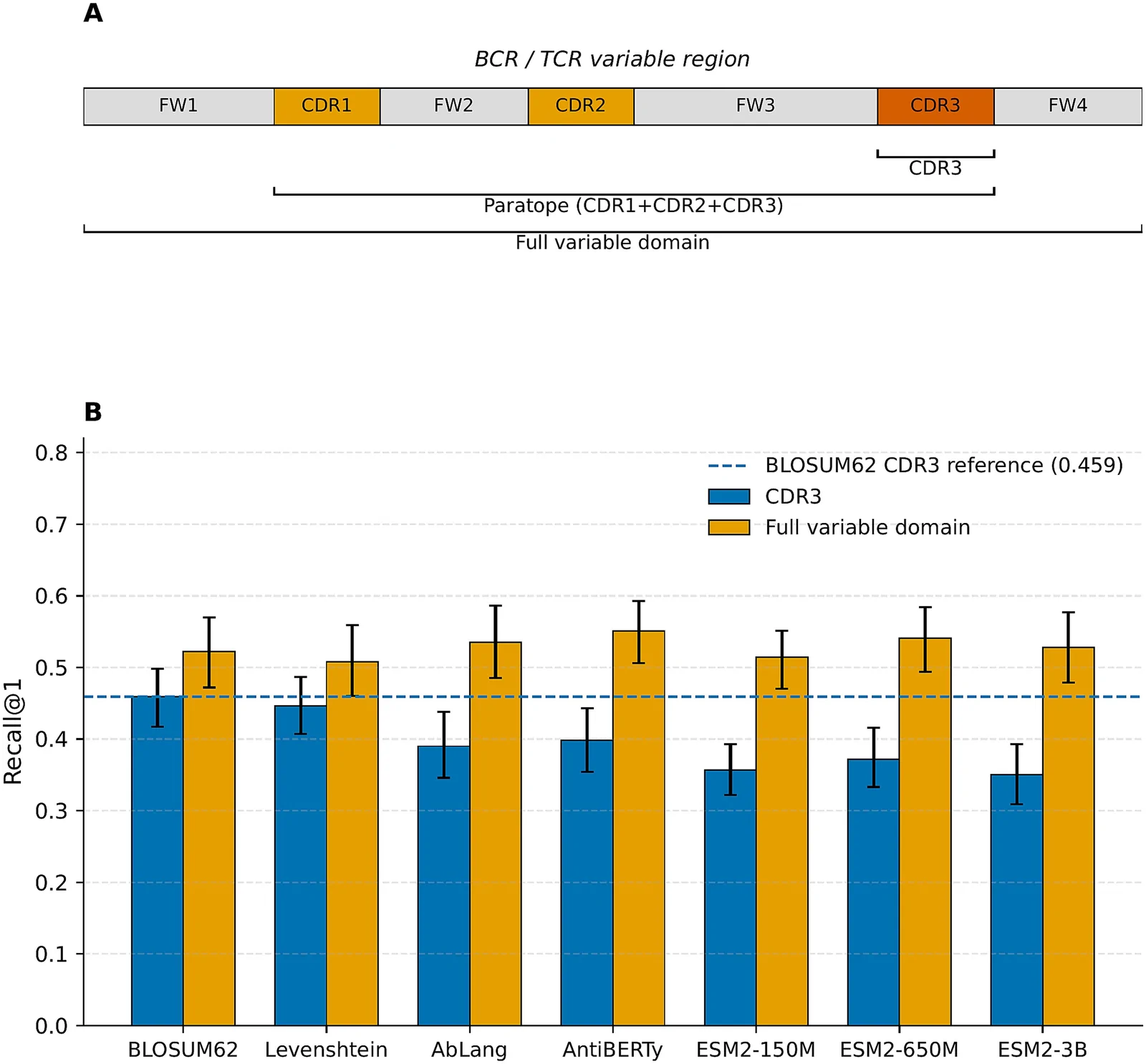
Benchmark design and BCR retrieval by representation. (A) Schematic of the receptor variable region (BCR heavy chain or TCR beta chain) with CDR3, paratope (CDR1 + CDR2 + CDR3), and full variable domain representation brackets. The clone-aware split groups sequences into connected components at CDR3 similarity ≥0.95. (B) BCR retrieval for alignment methods (BLOSUM62, Levenshtein), antibody-specific PLMs (AbLang, AntiBERTy), and the ESM2 family at the CDR3-only and full variable domain representations (the all-CDR paratope input, where alignment peaks, is in Table 1 and Fig. 3); dashed line, 20-seed BLOSUM62 CDR3 reference (R@1 = 0.459). At the CDR3, the two alignment methods lead all PLMs; at the full variable domain several methods converge near R@1 ≈ 0.51–0.55 (including the antibody-specific AbLang 0.535 and AntiBERTy 0.551). Error bars: 95% nested-bootstrap CI across 20 seeds.

**Figure 3:**
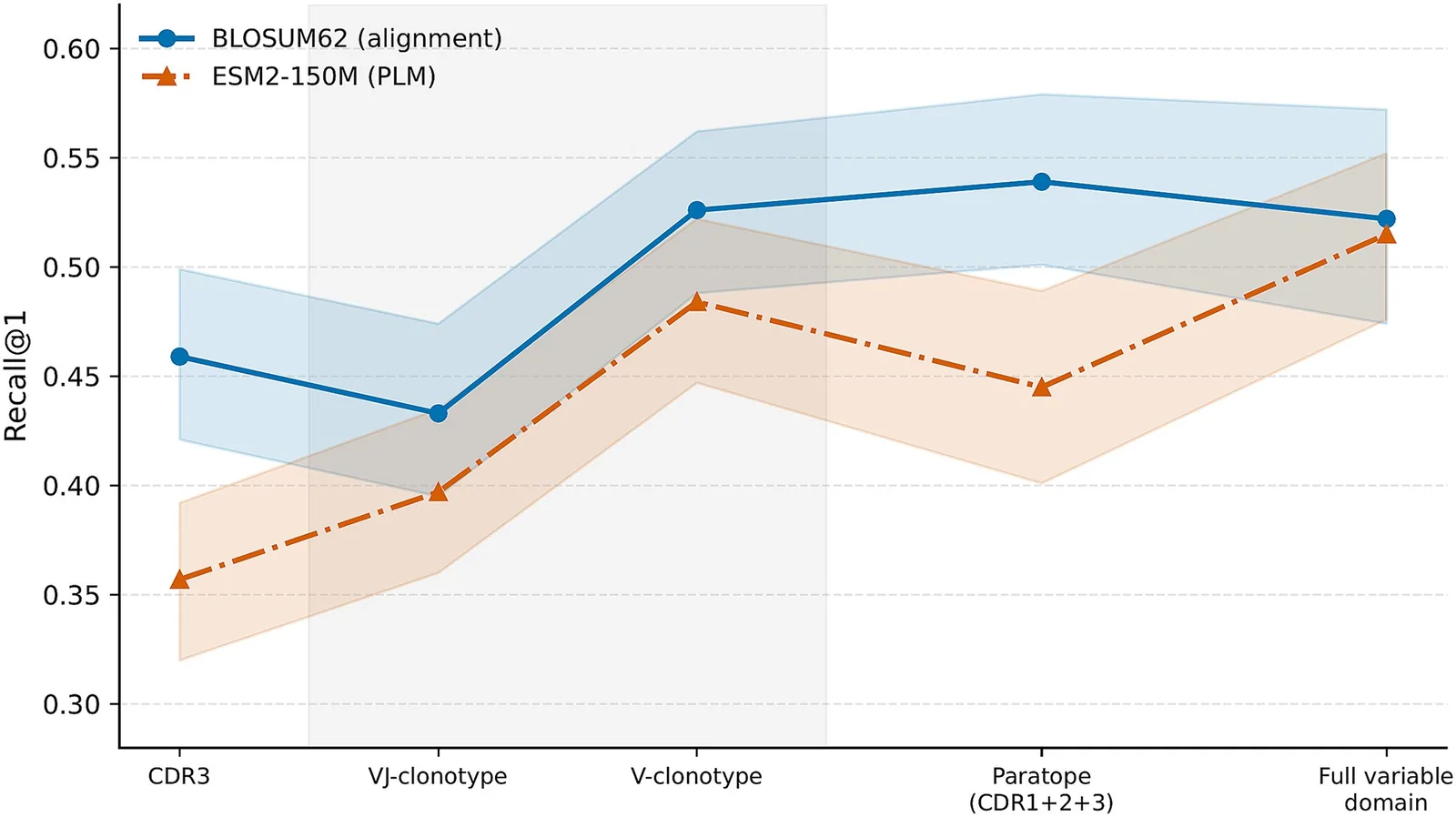
BCR retrieval across the representation ladder. BCR Recall@1 across the ladder in defined order (CDR3, VJ-clonotype, V-clonotype, paratope, and full variable domain; 20-seed nested bootstrap). The gray band marks the two clonotype-retrieval variants (VJ-clonotype, V + J; V-clonotype, V only), which keep the CDR3 input but add a germline-gene filter (subsection Receptor representations); the other representations vary input content. These are two distinct axes (input content versus retrieval constraint) and should not be read as a single monotonic trend along the *x*-axis. BLOSUM62 leads at the CDR3 input and peaks at the paratope, gaining nothing from the framework; ESM2-150M trails alignment from the CDR3 to the paratope and reaches parity only at the full variable domain (germline-reversion analysis in subsection At full length, PLMs and alignment converge through agermline-associated signal; per-representation values with 95% CIs in Supplementary Table S3). Shaded bands: 95% nested-bootstrap CI.

Neither model scale, domain specialization, nor newer architectures closed the gap. No ESM2 model matched alignment on any dataset; antibody-specific AntiBERTy (0.398 at BCR CDR3, 6.1 pp below BLOSUM62), AbLang, and the more recent AntiBERTa2 (0.345) and IgBert (0.383), released in 2023 and 2024, likewise fell short at the CDR3, as did TCR-BERT. ESM-C-300M performed similarly to ESM2-150M, remaining 7.1–9.9 pp below BLOSUM62 across datasets (Supplementary Table S15). The two newer antibody-specific models reached alignment only at the full variable domain (0.531 and 0.514), showing the same full-length convergence pattern as other PLMs; newer antibody-specific architectures therefore did not close the CDR3 gap.

The deficit is also a property of the embeddings rather than the retrieval metric. A linear probe trained on frozen CDR3 embeddings likewise failed to match alignment; correcting embedding anisotropy (the embeddings’ skewed geometry) improved R@1 by at most 3–5 pp, and the deficit persisted under an alternative distance metric but disappeared for full-variable-domain embeddings despite their similar geometry (Supplementary Note S2.6; Fig. S11). The deficit was largest for the CDR3s that ESM2 considered most unusual, yet did not track divergence from germline, leaving the antigen-discriminating feature missed by the embeddings unresolved.

The one clear exception, the most heavily hypermutated antibodies, is examined in subsection A directional exception: PLMs lead on the most hypermutatedantibodies.

### At full length, PLMs and alignment converge through a germline-associated signal

At the full-variable-domain representation, the CDR3 performance hierarchy disappeared. The five primary benchmark methods (two alignment methods and three ESM2 models) fell within a ~3.3-pp range (0.508–0.541; Table 1), with overlapping 95% confidence intervals. Practical equivalence testing (TOST; subsection Clone-aware splitting, seeds and statistical analysis) placed most comparisons within the prespecified Δ = 0.05 R@1 margin, including ESM2-150M versus BLOSUM62 (mean difference −0.005 across seeds). The one exception was ESM2-650M versus Levenshtein: its mean difference (+0.034) lay within the margin, but its confidence interval extended just beyond Δ = 0.05, so equivalence was not formally established. The CDR3 ordering therefore effectively reversed, with the best ESM2 model slightly exceeding the weaker alignment baseline (Fig. 2; Supplementary Table S4).

The representation ladder shows how this convergence arises (Fig. 3). At the CDR3, alignment exceeded ESM2-150M by 10.2 pp. The clonotype representations (VJ-clonotype and V-clonotype) mainly changed reference coverage rather than candidate ranking: the stricter VJ filter drove every method to a common floor by eliminating many valid matches, whereas the V-only filter improved retrieval where coverage permitted (IEDB BCR and McPAS-TCR, but not VDJdb). Alignment maintained a modest advantage across these intermediate representations (Supplementary Table S1). V-clonotype is effectively within-V-gene CDR3 retrieval: it restricts each query to same-V candidates and ranks them by the CDR3. Alignment still led the PLM there (BCR BLOSUM62 0.526 versus ESM2-150M 0.483), showing that the CDR3 gap persists after V-gene composition is controlled.

Adding CDR1 and CDR2 to form the complete paratope improved both method families by ~8 pp, but PLMs still trailed alignment by 9–13 pp. Only after adding the conserved framework did their trajectories diverge: PLMs improved further, whereas alignment declined slightly, bringing the two approaches to practical equivalence. Alignment therefore peaks at the paratope, whereas PLMs reach parity only at full length.

This pattern indicates that the strongest antigen-discriminating signal available to alignment is concentrated in the CDRs. Alignment achieved its highest performance on the paratope (the best BCR result in Table 1, Levenshtein R@1 = 0.550), and adding the framework slightly reduced performance, consistent with dilution of CDR differences without additional detectable antigen-discriminating information. Part of the CDR3-to-paratope gain is itself germline-derived through CDR1 and CDR2, as shown below. In contrast, every PLM, including AntiBERTy and AbLang (Supplementary Table S15), benefited from the framework, consistent with the reported germline bias of antibody language models [5].

Only PLMs benefited from the framework. The method × representation interaction was positive in all 24 method × model-size × dataset comparisons (paired Hedges’ *g* 0.60–1.95). Because these comparisons are not independent, we treated the four databases as the replication units; all four showed the same interaction (Supplementary Note S2.2). By itself, however, this interaction does not establish germline dependence, since framework sequence could contain antigen-discriminating information accessible only to PLMs. The following analyses distinguish these possibilities.

We therefore describe the general phenomenon as germline dependence. In the narrower context of this benchmark, the dependence can function as a shortcut: zero-shot PLMs close their CDR3 deficit by leveraging conserved germline-associated sequence in the framework, whereas alignment, already saturated on the CDRs, does not benefit from it and declines slightly. Full-variable-domain retrieval is not determined solely by germline identity, as performance substantially exceeds a V-gene-only baseline (below). The same conclusion was obtained with alternative embedding-pooling strategies (Supplementary Note S2.2).

#### Input format versus germline content

A natural concern is that full-length parity could reflect input “format” rather than germline “content”, because the full variable domain may better match the PLM’s pretraining distribution. We embedded the same full-variable-domain sequence once and compared four pooling strategies: mean, CLS, CDR-only, and framework-only (Supplementary Note S2.2). All four were similar, with overlapping 95% confidence intervals and no systematic CLS advantage. Because this control does not separate pretraining-distribution effects from sequence content, the germline-reversion test below holds input length and format fixed while changing framework sequence.

#### Germline-reversion test

We replaced the antibody framework with its inferred germline sequence while retaining native CDRs, and separately replaced CDR1 and CDR2 with germline sequences while retaining the native framework (20 paired seeds). For ESM2-150M, germline framework alone accounted for approximately two-thirds of the PLM improvement from the paratope to the full variable domain (*P* < 10^−5^). Reverting CDR1 and CDR2 produced no detectable loss, while framework somatic mutations contributed little overall and no measurable benefit outside HIV-1 antibodies (subsection A directional exception: PLMs lead on the most hypermutatedantibodies). BLOSUM62 remained essentially unchanged throughout. The same pattern was reproduced in SAbDab (Supplementary Note S2.5). Together, these results provide strong evidence that the full-variable-domain gain is driven substantially by conserved germline-associated framework sequence rather than framework somatic mutations.

#### V-gene oracle

Across all full-variable-domain methods, 91.5%–99.2% of top-ranked retrievals shared the query’s V-gene family, versus ~34% expected by chance (Supplementary Fig. S1, Table S11 and Note S2.4). Yet, explicitly providing the correct V-gene family did not improve retrieval. A two-stage oracle (true V-gene family followed by full-variable-domain BLOSUM62) achieved R@1 = 0.507 (95% CI 0.464–0.550), essentially identical to full-variable-domain BLOSUM62 alone (0.513 on the V-assignable subset). Knowing only the V gene, by contrast, gives R@1 = 0.190 (95% CI 0.181–0.200). Full-variable-domain alignment therefore already captures essentially all retrieval signal associated with V-gene identity, while V-gene information alone is insufficient. We regard the oracle as a consistency check rather than decisive evidence, with the germline-reversion experiment carrying primary weight.

Each analysis has limitations (Supplementary Note S2.2), so we present the shortcut interpretation as a working model rather than a proven mechanism. It is nevertheless supported by three additional observations: germline-V enrichment in native TCR retrieval (subsection Generality and robustness), loss of SCEPTR’s apparent advantage after clonotype filtering (subsection Generality and robustness), and embedding-geometry controls showing that the CDR3 deficit reflects the embeddings rather than the similarity metric (Supplementary Note S2.6).

### A directional exception: PLMs lead on the most hypermutated antibodies

The overall convergence at the full variable domain concealed a clear antigen-specific exception (Supplementary Fig. S7; Table S8). BLOSUM62 outperformed ESM2-150M for most antigens, including *Plasmodium* CSP, SARS-CoV-2 spike, and influenza HA (by 7–10 pp), whereas HIV-1 envelope showed the opposite pattern, with ESM2 leading by 21.2 pp. HIV-1 was therefore the only major antigen for which a zero-shot PLM clearly outperformed alignment.

Because broadly neutralizing HIV-1 antibodies are extensively somatically mutated and well represented in public databases, we tested whether this advantage reflected pretraining leakage. A BLAST audit identified seven CDR3s matching documented broadly neutralizing antibodies in UniRef90, but excluding them changed the ESM2 advantage by only +1.1 pp. The leak-free HIV-1 subset still favored ESM2 by 22.2 pp (Supplementary Note S2.3), arguing against memorization.

Stratifying antibodies by SHM showed an association between germline divergence and PLM performance but did not support a general SHM-dependent mechanism. ESM2 led by 7.0 pp in the highest-SHM bin, yet this crossover was driven largely by HIV-1 envelope and disappeared within influenza HA and other non-HIV antigens at comparable SHM levels (Supplementary Note S2.3; Table S9). We therefore interpret this as a directional, hypothesis-generating exception rather than evidence for a general SHM mechanism. The data support an association between strong germline divergence and improved zero-shot PLM performance, but not the mechanism underlying it (see also Supplementary Note S2.5).

### Generality and robustness

#### TCR and paired chains

The TCR representation ladder mirrored the BCR pattern (Supplementary Table S1). Because full-length TCRβ chains were reconstructed from germline templates (subsection Datasets and antigen labels), these results serve primarily as a constructive control; mechanistic interpretation instead rests on clonotype-filter convergence and native-TCR V-gene enrichment. In the paired VDJdb analysis (Table 2), alignment again peaked at the paratope, whereas ESM2 reached parity only at full length. Requiring both alpha- and beta-chain V and J genes to match left almost every query without an eligible reference, collapsing all methods to the same floor (paired VJ-clonotype R@1 = 0.122–0.123). In native TCRs, 67%–70% of CDR3 top-ranked hits shared the query’s TRBV gene family for both alignment and ESM2, versus 7.8% expected by chance (~9-fold enrichment). TCRdist remained strongest (R@1 = 0.515 [0.489, 0.541]) and benefited most from paired chains (+8.7 pp versus +3.7 pp for BLOSUM62); because it explicitly scores germline-derived CDR1, CDR2, and CDR2.5 together with CDR3, part of its advantage is germline-informed.

**Table 2:** TCR paired-chain ladder (20 seeds, VDJdb). Expected R@1 for 5 benchmark methods across the paired ladder in defined order (10 fixed epitope labels, clone-aware split). The input representations (CDR3, paratope, and full variable domain) are interleaved with the two clonotype-retrieval variants (VJ-clonotype, V + J on both chains; V-clonotype, V on both chains), which keep the paired CDR3 input but add a germline-gene filter (subsection Receptor representations). Bold marks each method’s highest value among the input representations, meaning the best performance for each method. The full-variable-domain paired input uses germline-reconstructed full variable domains and is interpreted as directional (subsection Datasets and antigen labels). tcrdist3 is a single value because tcrdist3 computes one IMGT-weighted distance over CDR1/CDR2/CDR2.5/CDR3 of both chains, independent of the input ladder; it is reported alongside the full-variable-domain paired result for direct comparison.

Method	CDR3 paired	VJ-clonotype paired^a^	V-clonotype paired^a^	Paratope paired	Full variable domain paired
Levenshtein	0.414	0.123	0.180	**0.443**	0.437
BLOSUM62	0.436	0.123	0.179	**0.459**	0.437
ESM2-150 M	0.352	0.123	0.178	0.395	**0.424**
ESM2-650 M	0.349	0.122	0.178	0.396	**0.427**
ESM2-3B	0.363	0.123	0.180	0.399	**0.430**
*tcrdist3*	—	—	—	—	**0.515**

^a^Clonotype variants (subsection Receptor representations): reference candidates are restricted to those sharing the query’s germline genes on both chains (V and J for VJ-clonotype; V only for V-clonotype), then ranked by the paired CDR3; a query with no same-clonotype reference is a miss. Requiring a paired match is strict, so VJ- and V-clonotype paired fall far below CDR3; under either filter all five methods are statistically indistinguishable (full discussion in subsection Generality and robustness; 95% CIs in the released Table_S1_Full_CI.csv).

#### Pairing

Antibody heavy–light pairing left all five methods statistically indistinguishable on both IEDB (R@1 0.52–0.54) and SAbDab pairs (0.35–0.36; Supplementary Table S5). Pairing thus had an asymmetric effect: it substantially improved TCR retrieval (especially TCRdist) but contributed little for antibodies, which we do not interpret further given the limited paired-antibody datasets (subsection Limitations).

#### SCEPTR, a contrastively pretrained TCR language model

SCEPTR [3], which jointly encodes the V gene and CDR3 through a TCR-specific contrastive objective, was the only PLM to match alignment at the CDR3. Under the same clone-aware benchmark and common gene-mappable query set, it achieved R@1 = 0.457 on McPAS-TCR and 0.333 on VDJdb, matching TCRdist and exceeding BLOSUM62 by 2–6 pp, while ESM2 remained 11–18 pp lower (Supplementary Table S14). However, restricting retrieval to receptors sharing the query’s V or V/J gene removed this advantage, with SCEPTR converging with or falling below BLOSUM62 (Supplementary Table S1). Even incorrect top-ranked matches were enriched for the query V-gene family, indicating strong prioritization of germline identity. On paired alpha–beta chains, SCEPTR likewise converged with generic alignment (0.468 versus 0.467), leaving only TCRdist (0.490) ahead on the common subset. Thus, SCEPTR reaches the same germline-informed frontier rather than clearly adding antigen-discriminating signal beyond alignment. Because its original benchmark included VDJdb-derived data, McPAS-TCR provides the more independent validation.

#### TCR2vec and CDR3vec, similarity-pretrained TCR models

TCR2vec [29] was evaluated in its CDR3-only (CDR3vec) and full-length V–CDR3–J variants. CDR3vec trailed alignment at the CDR3 (McPAS-TCR R@1 = 0.376 versus BLOSUM62 0.394; VDJdb 0.300 versus 0.319). Full-length TCR2vec matched alignment on McPAS (0.420) and exceeded CDR3vec by 4.4 pp, but restricting retrieval to same-V candidates erased this advantage, with TCR2vec, CDR3vec, and BLOSUM62 converging at 0.497, 0.514, and 0.513, respectively. On VDJdb, TCR2vec remained below alignment (0.311 versus 0.319). Thus, its full-length gain over CDR3vec is largely attributable to germline V context rather than improved CDR3 discrimination. The 128-dimensional variants and TCRdb-pretrained TCR2vec showed the same pattern. In paired alpha–beta evaluation, both remained at the alignment frontier (TCR2vec 0.420, CDR3vec 0.458 versus BLOSUM62 0.468), with only paired-native SCEPTR ahead (0.498; Supplementary Table S15).

#### Robustness

The CDR3 advantage of alignment was robust across CDR3 length strata, most evaluable BCR antigen classes and VDJdb epitope labels (Supplementary Fig. S3; Supplementary Table S7). Varying the clone threshold from 0.85 to 0.99 preserved both the CDR3 advantage (10–13 pp) and practical equivalence at full length (Supplementary Fig. S4; Table S6). Across receptor classes, databases, chain configurations, and clone definitions, alignment extracted more antigen-relevant information from the CDR3, whereas PLMs reached parity only after conserved germline information was introduced through the full variable domain. We adapted the CoV-AbDab antibody-discovery setting used by Wang et al. [33] to a zero-shot neutralizer-versus-binder-only task so that it could be evaluated under the same protocol as the main benchmark. Using a balanced 4000-antibody working set drawn from 10 920 SARS-CoV-2 binders and the same clone-aware expected-R@1 protocol, alignment led every PLM at the CDRH3 (BLOSUM62 R@1 = 0.607, AUROC = 0.651; ESM2-150M = 0.567/0.583), while the methods converged at the full variable domain (Supplementary Note S2.7; Table S16).

## Discussion

Using a clone-aware benchmark spanning four immune-receptor databases, we found that classical sequence alignment consistently outperformed frozen zero-shot PLMs for CDR3 retrieval, whereas the difference largely disappeared at the full variable domain. Together with the germline-reversion and V-gene analyses, these findings support germline dependence as a major contributor to full-length convergence. In this benchmark, that dependence can provide a shortcut to improved retrieval without demonstrating that PLMs have learned more antigen-specific information from the CDRs (Fig. 4).

**Figure 4:**
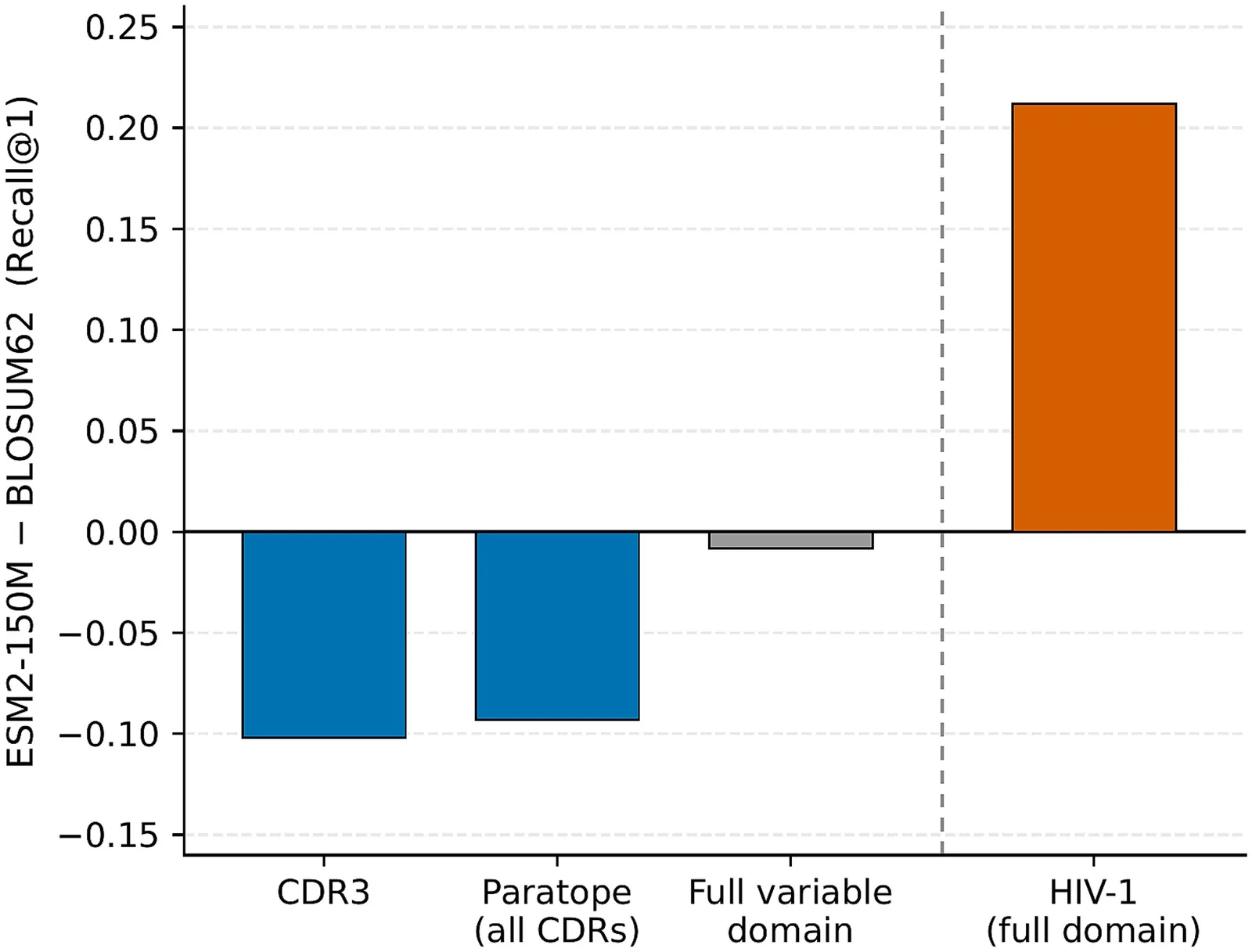
The germline axis in one view (BCR; illustrative). ESM2-150M minus BLOSUM62 Recall@1 across four settings grouped into three germline regimes; bars are colored by which family leads. Same antibodies, increasing germline content of the input (left of the divider): with CDR-level input and no framework, alignment leads at CDR3 only (CDR3, −0.10) and at all CDRs (paratope, −0.09); adding the conserved germline framework at the full variable domain (−0.01, TOST-equivalent) closes the gap, the visual signature of germline dependence. Different antibodies, same full-variable-domain input (right of the divider): on the heavily hypermutated, far-from-germline HIV-1 envelope subset the PLM leads (full variable domain, +0.21; leak-free +0.22, subsection A directional exception: PLMs lead on the most hypermutatedantibodies). The first three bars vary receptor representation for one antibody set; the fourth changes the antibody set (not the representation), so it is offset. The regional axis (framework versus CDR3) locates where the PLM trails, but the within-CDR3 mechanism is not captured by a model-independent germline distance and remains open (Supplementary Note S2.6). This panel summarizes the BCR ESM2-150M-vs-BLOSUM62 contrast; full cross-database and cross-size evidence is in Table 1 and subsection Generality and robustness. Values are 20-seed nested-bootstrap means (Table 1; subsection A directional exception: PLMs lead on the most hypermutatedantibodies, Supplementary Note S2.3).

We use germline dependence to describe the general observation that full-length PLM retrieval relies strongly on germline-associated context. We reserve shortcut for the narrower benchmarking mechanism in which a relatively low-dimensional, readily available signal, particularly germline V-gene identity, improves full-length retrieval without demonstrating additional higher-order antigen-specific information from the CDRs. This terminology does not imply that germline information is an artifact or biologically unimportant.

Our controls support this interpretation from complementary directions. The V-gene oracle did not exceed full-length alignment (0.507 versus 0.513), whereas germline framework reversion reproduced most of the PLM full-length gain while retaining native CDRs. Thus, conserved germline-associated context accounts for a substantial fraction of the PLM gain, but V-gene identity alone does not determine retrieval and the framework may contain other useful information. The key point is that full-length improvement cannot by itself be interpreted as improved CDR-level antigen discrimination. This distinction matters because germline genes genuinely influence antigen recognition, including well-characterized antibody classes such as VH1-69 in influenza [34] and VH1-2 in HIV-1 CD4-binding-site antibodies [35]. In our benchmark, however, PLMs reached alignment parity only after conserved germline-associated framework sequence became available. Germline-reversion and V-gene analyses support the same interpretation, while TCRdist and the contrastively pretrained SCEPTR also derive part of their advantage from germline-derived information. The CDR3 deficit and full-length convergence therefore appear consistent with a common representational limitation: frozen embeddings exploit conserved germline context effectively but show no corresponding advantage in extracting antigen-discriminating information from highly diverse junctional CDR3s.

A further question is whether this germline-associated signal is biological or partly a dataset-construction artifact: mutagenesis campaigns or single-study cohorts can generate many receptors for one antigen that share V and J genes by construction, rather than by binding, creating spurious associations [36]. Three features of our design make a purely constructed signal less likely. Clone-aware splitting assigns lineage and mutagenesis variants to a single partition, removing the most direct construction artifact from the leakage path. The V-gene oracle shows that V-gene identity alone is far from sufficient (R@1 0.19 versus 0.51 at full length), and the germline-reversion effect replicates across databases of different provenance (mutagenesis-rich IEDB and structure-derived SAbDab). We nevertheless cannot fully separate biological germline preference from cohort structure at the present label resolution, which remains a limitation.

The principal exception was HIV-1 envelope antibodies, the only major antigen class for which PLMs substantially outperformed alignment. These antibodies occupy one of the most highly mutated regions of antibody sequence space [37]. Although our data do not establish a causal role for somatic hypermutation, the exception occurs where receptors diverge furthest from germline, suggesting that zero-shot PLMs may be most useful for highly evolved antibody lineages rather than typical repertoires. This interpretation is specific to the HIV-1 setting examined here and should not be generalized to somatic hypermutation broadly. Because somatic hypermutation is unique to B cells, this regime is not expected for TCRs.

Our conclusions apply specifically to frozen zero-shot embeddings and do not imply that PLMs are intrinsically unsuitable for immune-receptor analysis. Fine-tuning, contrastive learning, or explicit incorporation of germline information may outperform alignment, as suggested by recent studies [3, 38, 39]. Our contribution is to establish the zero-shot baseline against which such methods should be evaluated and explain why full-length convergence can occur despite inferior CDR3 retrieval. The independent BCR-native SARS-CoV-2 neutralization analysis further suggests that this distinction extends beyond antigen-specific retrieval, although its representation-level interpretation remains subject to the task’s limitations.

The shortcut interpretation is not a claim that general PLMs are weak [5, 6], but a claim about the composition of the retrieval signal, and the same issue can arise in receptor-specific models. SCEPTR reaches alignment-level CDR3 retrieval, but its advantage disappears when retrieval is restricted to the same V-gene context. TCR2vec likewise shows a full-length gain consistent with germline-bearing context rather than demonstrably improved CDR discrimination [29]. Thus, receptor-specific models do not make the finding moot: even purpose-built models can gain substantially from the low-dimensional germline axis without demonstrating improved capture of the junctional signal distinguishing specificities.

More broadly, these findings illustrate a recurring challenge in biological machine learning: strong benchmark performance does not necessarily imply that a model has learned the intended biological signal. In immune-receptor retrieval, near-parity at full length can arise largely from germline-associated information rather than improved encoding of antigen-discriminating features. This is consistent with supervised antibody benchmarks showing that general PLMs help mainly low-specificity tasks, while high-specificity function depends on biological mechanism rather than model scale [33], with evidence that TCR embedding similarity can largely re-express sequence-space similarity [29], and with work showing that learned models can encode selection factors shaping receptor repertoires [40]. Distinguishing these signal sources will therefore be important for evaluating future immune-receptor representations and understanding what PLMs encode in the zero-shot setting.

### Practical implications

These findings motivate six recommendations for immune-receptor retrieval benchmarks (Supplementary Box S1): use clone-aware splitting; report tie-aware retrieval metrics; include a zero-shot sequence-alignment baseline; stratify performance by receptor representation and germline context; assess robustness across clone definitions and datasets; and report embedding provenance and model version.

For practice, the recommendations are straightforward (Table 3). For CDR3-only retrieval, BLOSUM62 or Levenshtein should remain the default zero-shot methods. For BCRs, alignment on the complete paratope provides the highest retrieval accuracy, whereas adding the framework does not further improve alignment. At the full variable domain, ESM2-150M and BLOSUM62 perform similarly overall, so method choice should depend on representation and biological setting rather than model scale alone. For paired TCR retrieval, TCRdist remains the strongest method. Random train–test splitting should be avoided because it produces optimistic retrieval estimates relative to the clone-novel target. Together, these recommendations provide a reproducible baseline: benchmark clone-aware, report the representation evaluated, and distinguish gains from germline-associated context from gains in antigen-specific representation. Recommendations are ranked by accuracy; the accuracy–speed trade-off is discussed below.

**Table 3:** Practical recommendations for zero-shot benchmarking.

Setting	Recommended zero-shot method	Rationale
CDR3-only BCR/TCR retrieval	BLOSUM62 or Levenshtein	Consistent CDR3 advantage over PLMs
Best zero-shot accuracy (BCR)	BLOSUM62 or Levenshtein on all CDRs (paratope)	Highest R@1 overall; adding framework sequence does not improve alignment performance
Full variable domain BCR, mean-pooled ESM2 available	BLOSUM62 or ESM2-150M	Comparable aggregate R@1; inspect antigen and germline strata
Paired TCR chains	TCRdist (tcrdist3)	TCRdist (R@1 = 0.515) significantly exceeds every tested alignment and ESM2 variant (R@1 0.42–0.44); BLOSUM62, Levenshtein, and the three ESM2 sizes are mutually comparable
Any benchmark evaluation	Clone-aware splitting	Random splits yield R@1 15–28 pp higher

Recommendations are ranked by accuracy.

Although alignment generally provides the highest zero-shot accuracy, PLM-based retrieval has an important computational advantage: embeddings are computed once and reused, whereas alignment requires pairwise comparisons for every query–reference pair. Embedding-based retrieval may therefore remain preferable for very large reference databases where search speed outweighs modest differences in retrieval accuracy.

### Limitations

This study has several limitations.

First, the available antigen-label space remains limited. Each benchmark contains only 5–10 antigen or epitope labels, and protein-level BCR annotations may group antibodies targeting distinct epitopes. Although the principal findings replicated across both protein- and peptide-level datasets, broader epitope-resolved benchmarks would better distinguish germline-associated from antigen-specific retrieval. The abundant-label subsets may also retain study, lineage, or germline structure (receptors annotated to one antigen may overrepresent particular cohorts or V genes), which our clone-aware splitting and V-gene oracle mitigate but cannot fully eliminate. Thus, some germline-associated signal may reflect dataset-specific structure in addition to biologically relevant information.

Second, the apparent PLM advantage among highly mutated antibodies was driven largely by HIV-1 envelope. Because additional highly mutated antigens are scarce, we could not separate somatic hypermutation from antigen identity. This result should therefore not be interpreted as evidence that PLMs generally outperform alignment for highly mutated antibodies.

Third, full-length TCR beta sequences were reconstructed from germline templates rather than observed directly. Accordingly, the strongest mechanistic evidence for the shortcut interpretation comes from native antibody analyses, whereas the TCR interpretation relies primarily on clonotype filtering, representation effects, and germline enrichment in native TCR retrieval.

Finally, our conclusions are limited to frozen, last-layer zero-shot embeddings. We assessed four pooling strategies (mean, CLS, CDR-only, and framework-only) and several receptor-specific and recent architectures (AntiBERTa2, IgBert, TCR2vec, and SCEPTR), but did not systematically evaluate intermediate layers, fine-tuning, contrastive learning beyond SCEPTR, joint receptor–antigen models, or the full and rapidly expanding family of newer PLMs. Likewise, although robustness analyses suggest that alternative retrieval metrics, similarity metrics, or clone definitions would not alter the principal conclusions, these remain methodological choices rather than biological ground truth.

### Future directions

Future work should extend these analyses to larger epitope-resolved datasets, particularly native paired full-length receptors and highly mutated antibodies beyond HIV-1. Germline-aware, contrastively trained, and fine-tuned PLMs should then be evaluated across the same representation hierarchy to determine whether they overcome the CDR3 limitation observed in the zero-shot setting.

More fundamentally, our results narrow the remaining question. Because the CDR3 deficit was not eliminated by the similarity metrics, embedding representations, or germline-divergence analyses tested here, the critical issue is which junctional features frozen PLMs fail to encode and whether those features can be recovered through improved pretraining or task-specific adaptation.

**Key Points**

- Classical sequence alignment consistently outperformed frozen protein language models (PLMs) for CDR3 immune-receptor retrieval across four datasets.
- PLMs reached parity only at the full variable domain after conserved germline-derived sequence was introduced, revealing strong germline dependence and a potential shortcut in benchmark performance.
- Frozen PLMs exceeded alignment only for highly mutated HIV-1 antibodies.
- The receptor-specific PLMs that matched alignment at the CDR3, SCEPTR, and TCR2vec, did so while additionally encoding germline V-gene information, rather than by demonstrably better CDR modeling.
- Random train–test splitting reported accuracy 15–28 percentage points higher than clone-aware splitting owing to clonal leakage; clone-aware splitting should be standard.

## Supplementary Material

Supplementary_Material_bbag614

## Data Availability

The benchmark framework, evaluation scripts, statistical analysis code, and download scripts are available at https://github.com/NidRUAwake/immune-embedding-benchmark. The four source databases are used under their respective licenses (IEDB, CC-BY-4.0; VDJdb, AGPL-3.0; SAbDab, CC-BY; McPAS-TCR, per the McPAS-TCR website terms) and are not redistributed as raw dumps; we instead point to each original source and the exact version used (IEDB downloaded 2026-05-09; VDJdb 2025-12-29 release; SAbDab downloaded 2026-05-05; McPAS-TCR standardized 2026-05-19). The BCR-native validation additionally used CoV-AbDab [41] (2024-02-08 release, downloaded from the OPIG web server), freely available under its terms of use. VDJdb’s 2025-12-29 release is byte-reproducible from its GitHub release asset; because IEDB, SAbDab and McPAS-TCR serve only their current release, we additionally provide the license-permitted processed inputs together with MD5 checksums for every input file to enable exact reproduction. Pre-computed embeddings are not included due to file size but can be reproduced using the provided embedding scripts. Pretrained model weights for ESM2 and TCR-BERT are obtained from HuggingFace; AbLang and AntiBERTy weights are bundled in their respective ablang and antiberty Python packages (the original HuggingFace repositories are no longer available; Supplementary Methods), all under their respective licenses.
